# Web-Based Alcohol and Sexual Assault Prevention Program With Tailored Content Based on Gender and Sexual Orientation: Preliminary Outcomes and Usability Study of Positive Change (+Change)

**DOI:** 10.2196/23823

**Published:** 2022-07-22

**Authors:** Amanda K Gilmore, Ruschelle M Leone, Daniel W Oesterle, Kelly Cue Davis, Lindsay M Orchowski, Viswanathan Ramakrishnan, Debra Kaysen

**Affiliations:** 1 Department of Health Policy & Behavioral Sciences School of Public Health Georgia State University Atlanta, GA United States; 2 Department of Psychological Sciences Purdue University West Lafayette, IN United States; 3 Edson College of Nursing and Health Innovation Arizona State University Tempe, AZ United States; 4 Department of Psychiatry & Human Behavior Alpert Medical School of Brown University Providence, RI United States; 5 Medical University of South Carolina Charleston, SC United States; 6 Department of Psychiatry & Behavioral Sciences Stanford University Stanford, CA United States

**Keywords:** sexual assault prevention, alcohol, college students, sexual and gender minorities

## Abstract

**Background:**

Alcohol use and sexual assault are common on college campuses in the United States, and the rates of occurrence differ based on gender identity and sexual orientation.

**Objective:**

We aimed to provide an assessment of the usability and preliminary outcomes of *Positive Change* (*+Change)*, a program that provides integrated personalized feedback to target alcohol use, sexual assault victimization, sexual assault perpetration, and bystander intervention among cisgender heterosexual men, cisgender heterosexual women, and sexual minority men and women.

**Methods:**

Participants included 24 undergraduate students from a large university in the Southwestern United States aged between 18 and 25 years who engaged in heavy episodic drinking in the past month. All procedures were conducted on the web, and participants completed a baseline survey, *+Change*, and a follow-up survey immediately after completing *+Change*.

**Results:**

Our findings indicated that *+Change* was acceptable and usable among all participants, despite gender identity or sexual orientation. Furthermore, there were preliminary outcomes indicating the benefit for efficacy testing of *+Change*.

**Conclusions:**

Importantly, *+Change* is the first program to target alcohol use, sexual assault victimization, sexual assault perpetration, and bystander intervention within the same program and to provide personalized content based on gender identity and sexual orientation.

**Trial Registration:**

ClinicalTrials.gov NCT04089137; https://clinicaltrials.gov/ct2/show/NCT04089137

## Introduction

### Background

Alcohol use and sexual assault are widespread problems on college campuses in the United States [1,2], and the rates of occurrence differ based on gender identity and sexual orientation [3,4]. A total of 52.5% of college students used alcohol in the past month, and 33% engaged in heavy episodic drinking (>4 drinks for females and >5 drinks for males in <2 hours) [1,5]. Individuals who identify as a sexual or gender minority (lesbian, gay, bisexual, trans, queer or questioning [LGBTQ]) engage in alcohol misuse at higher rates than their heterosexual and cisgender counterparts [3]. Similarly, sexual assault is common among college students, with students who identify as cisgender heterosexual women and LGBTQ both experiencing the highest rates of sexual assault [4]. Despite alcohol interfering with sexual assault victimization risk perception, sexual assault victimization resistance [6] and bystander behavior [7], as well as increasing the risk for sexual assault perpetration [8], no intervention has targeted alcohol use and sexual assault victimization, perpetration, and bystander intervention in a single intervention. This is problematic, given that sexual assault is a multifaceted issue, and providing prevention for one component of sexual assault (victimization, perpetration, or bystander intervention) cannot address the full scope of sexual assault. Furthermore, providing one prevention component to men (ie, perpetration prevention) may send the message that men are not victims of assault or that it is not necessary to address all 3 components. Targeting multiple, related health issues, such as alcohol and sexual assault, is more effective than targeting them separately [9,10]. Furthermore, no program has provided tailored feedback for cisgender heterosexual men, cisgender heterosexual women, and LGBTQ students, despite their differential risk and differential risk factors. Therefore, this study presents the usability of a novel social norms–based intervention, *Positive Change* (*+Change)*, which targets alcohol and sexual assault (victimization, perpetration, and bystander intervention) and is tailored based on gender identity and sexual orientation.

### Alcohol Use and Sexual Assault Among LGBTQ Individuals

Individuals who identify as LGBTQ are not a homogeneous group, and the drinking patterns of the LGBTQ community are, on average, equal to or greater than their cisgender heterosexual peers [11-15]. For example, one national study in the United States found that individuals who identify as lesbian or bisexual women and gay or bisexual men were 3.81 and 1.76 times more likely, respectively, to engage in high-intensity drinking (>12 drinks in a single drinking episode), compared with those who identify as heterosexual [3]. Another study indicated that women who identify as lesbian and bisexual were 10.7 times more likely to drink compared with women who identify as heterosexual [16]. This discrepancy in drinking behavior has been replicated in college students who identify as LGBTQ [14,17]. Drinking to cope with minority stress [18,19] and social norms of bar culture among individuals who identify as LGBTQ may explain the disparities in alcohol use among LGBTQ men and women [12,13,20]. According to the minority stress model [18,19], individuals who identify as LGBTQ experience daily heterosexist and transphobic aggressions and microaggressions that cause compounded stress over time. According to the negative reinforcement model, alcohol can be used to cope with stress. Therefore, drinking to cope with minority stress and LGBTQ-specific drinking norms are essential to address in interventions targeting alcohol use for LGBTQ individuals.

Lifetime rates of sexual assault victimization among LGBTQ individuals are higher than their cisgender heterosexual counterparts, with 63% of LGBTQ individuals experiencing sexual assault victimization [21]. Lesbian and bisexual women experience sexual assault victimization at higher rates and experience more mental health symptoms after sexual assault victimization, including higher rates of posttraumatic stress disorder and depression [22,23] compared with their heterosexual counterparts. Gay and bisexual men have also reported high rates of sexual assault victimization. A recent study found that 67% of gay and bisexual men reported an adult sexual assault victimization experience, and 67% of those sexual assaults involved alcohol [24]. This rate of sexual assault victimization is higher than that when examining national data sets of men in the United States. For example, the National Intimate Partner and Sexual Violence Survey found that 24.8% of men experienced sexual assault victimization in their lifetime [25]. LGBTQ populations may be uniquely targeted for sexual assault because of heterosexism. Furthermore, perceived normative behaviors related to resisting sexual assault may differ based on gender and sexual orientation. For example, as LGBTQ populations are disproportionately targeted for violence, they may believe that their peers would not support them if they used active resistance strategies when targeted for sexual assault. Little is known about bystander behaviors among students who identify as LGBTQ, but it is anticipated that there may be unique barriers to engaging in bystander intervention as a member of the LGBTQ community, mostly because LGBTQ populations are disproportionately targeted for violence.

Despite the heightened risk for heavy episodic drinking and sexual assault victimization [24,26-29], men and women who identify as LGBTQ are often overlooked in heavy episodic drinking and sexual assault prevention programs. One exception is an assessment of an in-person bystander intervention, *Green Dot*, which has been tested in high schools, and secondary data analyses examined whether the program was effective for individuals who identified as a sexual minority [30]. This program was not specifically adapted to the unique needs of LGBTQ students; however, it was found that there were reductions in victimization and perpetration among heterosexual youth but not sexual minority youth. Therefore, it is essential to adapt interventions made to prevent sexual assault to LGBTQ populations. Furthermore, it is essential to address this large gap by including alcohol use and sexual assault victimization risk reduction content specific to students who identify as LGBTQ and this study presents an initial step toward this needed effort.

### Integrated Personalized Normative Feedback Interventions

The National Institute on Alcohol Abuse and Alcoholism Alcohol Intervention Matrix [31] recommends both personalized normative feedback and skills training as evidence-based interventions with low cost, high effectiveness, and high reach potential. Personalized normative feedback interventions target perceptions of normative drinking, which is the strongest predictor of alcohol use [32,33]. Specifically, personalized normative feedback interventions correct misperceptions of peer alcohol use by comparing one’s own use with actual peer use as well as comparing one’s perceptions of peer use with actual peer use. In a systematic review, 64% (25/39) of the trials found support for descriptive drinking norms as a mechanism of change in alcohol use interventions [34]. However, current approaches do not account for the unique drinking patterns of cisgender heterosexual women, cisgender heterosexual men, and LGBTQ men and women.

Social norms approaches for prevention allow for the prevention of cross-cutting behavior in an integrated manner as targeting social norms for multiple risk behaviors can be done within one theoretical framework. Furthermore, given that alcohol is associated with an increased risk for sexual assault victimization and perpetration and a decreased likelihood of engaging in a potential sexual assault situation as a bystander [7,8,35], it is essential to target alcohol use, sexual assault victimization, perpetration, and bystander intervention within 1 integrated program. This study included preliminary testing of *Positive Change (+Change)*, a multipronged personalized normative feedback intervention targeting alcohol use and sexual assault (victimization, perpetration, and bystander intervention) within 1 prevention program.

Sexual assault is still widely prevalent, and prevention efforts have not resulted in 0 perpetration rates; thus, feminist scholars emphasize harm reduction behaviors to reduce sexual assault risk while still placing the responsibility of the sexual assault solely with the perpetrator [36,37]. Theoretical models for sexual assault victimization risk reduction focus on providing skills for sexual assault risk perception and empowerment to resist sexual assault [37,38]. As social and psychological barriers can interfere with using active sexual assault resistance strategies, a social norms approach can be used to correct misperceptions of peer disapproval of using active resistance strategies [9]. Similarly, perpetration prevention can use a social norms approach [39,40] by targeting perceptions of peer rape supportive attitudes, beliefs, and behaviors that foster sexual assault perpetration [41]. This approach can also be used to target bystander intervention behaviors. Bystander intervention training encourages bystanders to engage in interventions when witnessing potential sexual assault situations. These programs are based heavily on the Social Norms Theory, as sexual assault perpetrators overestimate supportive peer attitudes toward sexual assault [42]. A recent meta-analysis of sexual assault bystander programs indicated that students who participated in these programs engaged in more bystander behaviors and had more prosocial attitudes, compared with those who did not [42]. Thus, a social norms approach combined with skills training can be a useful approach to address alcohol use and sexual assault prevention (victimization, perpetration, and bystander intervention) within 1 multipronged, comprehensive program.

Researchers have integrated sexual assault victimization risk, perpetration, and bystander intervention programs as a multipronged approach to prevent sexual assault on college campuses [43]; however, this integrated approach has not yet been implemented or tested. Furthermore, no program to date has targeted sexual assault prevention using this multipronged approach with integrated alcohol content. There are several advantages to addressing the needs of all college students within 1 program. First, it is costly for universities and time-consuming for students to provide 4 separate prevention programs. Second, excluding LGBTQ students from prevention programming is a form of heterosexism and may contribute to the continuing higher rates of alcohol and sexual assault among this population. Third, integrated intervention programming is more effective than providing separate interventions for related health behaviors [9].

### This Study

This study assessed the usability and acceptability of *+Change*, a multipronged program targeting alcohol misuse, sexual assault victimization risk, sexual assault perpetration, and bystander intervention among cisgender heterosexual men, cisgender heterosexual women, and LGBTQ men and women. It was hypothesized that *+Change* would have high usability and acceptability ratings. Furthermore, although the study was not powered or designed to detect differences in outcomes related to indicators of alcohol use, sexual assault victimization, sexual assault perpetration, and bystander intervention, these indicators were examined to determine whether there was a decrease in alcohol use risk and sexual assault risk after *+Change*.

## Methods

### Participants

A total of 30 participants consented to and were enrolled in the study, and 24 (80%) participants fully viewed the content and postintervention questionnaire and were included in the analyses for this manuscript. The final sample included 24 undergraduate students aged between 18 and 25 years who engaged in heavy episodic drinking in the past month. Participants were recruited from a large university in the Southwestern United States.

### Measures

#### Demographics

Participants completed items assessing age, race or ethnicity identity, year in college, and relationship status.

#### Gender Identity and Sexual Orientation

To assess gender identity, participants were asked the following question: “Understanding gender identity can be complex, which one category best describes your gender identity now?” Prior research has validated the use of this item to assess gender identity [44]. Responses included the following options: (1) female, (2) male, (3) transgender (female-to-male), (4) transgender (male-to-female), and (5) other. To assess sexual orientation, participants responded to the following question: “Understanding that sexual identity can be complex, which one category best describes your sexual identity now?” This item has been used in previous research among sexual minority individuals [45]. Response options included the following: (1) lesbian, (2) gay, (3) bisexual, (4) queer, (5) two-spirit, (6) straight or heterosexual, (7) questioning, (8) other, and (9) prefer not to answer. Participants responding that they identified as male and straight were placed into the cisgender heterosexual male group. Participants who responded that they were female and straight were placed in the cisgender heterosexual female group. Finally, participants who responded that their sexual orientation was any response other than straight or heterosexual or that their gender was anything other than male or female were labeled in this study as LGBTQ (no participants in the sample identified as transgender or other).

#### Usability and Acceptability

Participants were asked 2 questions about intervention helpfulness for themselves (“How helpful did you find the intervention content?”) and their peers (“How helpful do you think the intervention content would be for students at [your university]?”) and one question about how distressed they were by the intervention (“How distressing did you find the intervention content?”). Participants rated these items on a scale from 1 (very unhelpful or not at all distressing) to 7 (very helpful or very distressing). Participants also responded to 2 instruments assessing the intervention’s usability and functionality. To assess usability, participants responded to the Post-Study System Usability Questionnaire [46]. This 18-item instrument uses a Likert-type scale with response options ranging from 1 (strongly disagree) to 7 (strongly agree), where participants indicated their agreement with items such as “it was comfortable using this web-based intervention” and “it was easy to find the information I needed.” The Post-Study System Usability Questionnaire includes subscale scores assessing system usefulness, information quality, and interface quality.

#### Alcohol Use

The Daily Drinking Questionnaire [47] was used to assess participants’ alcohol consumption during a typical week. Participants were asked how many standard drinks they typically consume on each day of the week and then queried on the amount of time (ie, in hours) they typically consume that amount of alcohol. The Drinking Norms Rating Form [48] was used to assess normative perceptions of alcohol use among peers. This instrument assessed the perceived amount to which other students at their university in a particular group consume alcohol during a specific time frame for each day of the typical week. The peer groups assessed in this study included cisgender heterosexual women, cisgender heterosexual men, and LGBTQ, intersex, and asexual (LGBTQIA+) students at their university. The Injunctive Drinking Behaviors Scale [49] is a 15-item scale that was used to assess injunctive norms regarding the acceptability of drinking-related behaviors. Example items include rating how acceptable the typical student thinks it is to “drink shots” or to “drink alcohol every weekend.” Participants responded using a Likert-type scale, where they rated each behavior from 1 (unacceptable) to 7 (acceptable), with higher scores indicating increased levels of acceptability for each behavior. The contemplation ladder [50] assesses stages of change for alcohol use on a 0 (I have no thoughts of changing my drinking now) to 10 (I’m taking action to change [ie, cutting down]) scale.

#### Sexual Assault Victimization Risk

To assess the perceived risk of sexual assault victimization while intoxicated, participants were asked how likely they would be incapacitated by alcohol while engaging in unwanted sex. Participants responded using a 7-point Likert-type scale, with response options ranging from 1 (very unlikely) to 7 (very likely). Participants were also asked to estimate the percentage (0 to 100) of each group (cisgender heterosexual men, cisgender heterosexual women, and LGBTQIA+ students at their university) who have experienced sexual assault victimization since entering college.

#### Sexual Assault Perpetration Risk

The Stages of Change Scale [51-53] assessed the participants’ perceptions of sexual assault prevention efforts on campus. This instrument contains 8 items such as “I don’t think sexual assault is a big problem on campus” and “I am actively involved in projects to deal with sexual assault on campus.” The response options ranged from 1 (strongly disagree) to 4 (strongly agree), with higher scores indicating greater agreement with each item. The Illinois Rape Myth Acceptance Scale [54] was used to accept participant endorsement of 8 common rape myths on a Likert-type scale, with response options ranging from 1 (strongly disagree) to 4 (strongly agree), with high scores indicating greater agreement for each item. To assess the perceived risk for sexual assault perpetration, participants were asked how likely they would be to ask for verbal consent during sexual activity while drinking. To estimate the percentage of false reports at their university, participants were asked “What percent of sexual assaults are falsely reported at [your university]?” Participants were also asked how likely they would be to decide not to engage in sexual activity with someone who is drunk on a 6-point Likert-type scale, with response options ranging from 1 (not at all likely) to 6 (very likely).

#### Bystander Intervention

The Bystander Efficacy Scale [53] assessed participants’ confidence in performing prosocial behaviors related to the prevention of sexual violence. Specifically, the Bystander Efficacy Scale is an 18-item measure, which contains items where participants rate their confidence in performing behaviors such as “ask a friend/stranger if they need to be walked home from a party” and “speak up to someone who is making excuses for forcing someone to have sex with them.” Participants rated each of the 18 behaviors on a 0% (can’t do) to 100% (very certain) scale as to their confidence in performing the corresponding behavior, with high scores indicating greater levels of confidence.

### Procedure

Participants aged 18 to 25 years, who engaged in heavy episodic drinking in the past month, were recruited from a large university in the Southwestern United States using a random sample of students from the registrar by email. From a list of over 6000 students, 468 (7.8%) prospective participants were randomly selected to receive an email inviting them to participate in this study. Of those 468 invited to participate in the screening survey, 41 (88%) participants were eligible, and 30 (6.4%) participants were enrolled in the open pilot trial. Of these 30 participants, 24 (80%) completed the study procedures. The participants were capped to ensure equal recruitment of cisgender heterosexual women, cisgender heterosexual men, and LGBTQIA+ students. Participants completed consent procedures, a baseline survey, a social norms–based personalized feedback intervention (*+Change*), and a postintervention survey. All study procedures were performed on the web. They were compensated with US $25 for their participation.

### Ethics Approval

All study procedures were approved by the Georgia State University’s institutional review board (H2006) and participants consented to all study procedures before participating in the study.

### +Change Program Content

*+Change* included content from an integrated alcohol and sexual assault risk reduction program for women [9] and a web-based adaptation of a brief motivational interviewing personalized feedback protocol integrated with the men’s workshop for sexual assault perpetration and bystander intervention for men [41,55]. Given that the previous interventions included separate content for men and women and did not address the needs of LGBTQ men and women, new content was created for men’s victimization risk, women’s perpetration risk, and women’s bystander intervention skills training. Furthermore, content was created for the LGBTQ students. The intervention content underwent a rigorous process of intervention development where mockups were provided to college students (equal numbers of cisgender heterosexual men, cisgender heterosexual women, and sexual or gender minorities) and administrators for extensive feedback in interviews and focus groups, and iterative changes were made based on the feedback.

Participants received personalized feedback based on their answers to the baseline survey compared with a larger sample of college students at their university. Feedback was tailored by gender and sexual orientation (cisgender heterosexual men, cisgender heterosexual women, and LGBTQ individuals). *+Change* targeted heavy episodic drinking and sexual assault by integrating existing, theoretically driven [9,56-58], and evidence-based prevention initiatives delivered via a web-based platform: (1) social norms approach to reduce or prevent alcohol misuse, (2) programming to reduce sexual assault perpetration, (3) bystander intervention to all students tailored by gender and sexual orientation, and (4) sexual assault risk reduction programming tailored by gender and sexual orientation (Multimedia Appendix 1 [9,56-58]).

### Analytic Plan

Independent 2-tailed *t* tests were used to examine preintervention and postintervention differences. Given the small sample size, we examined both the trends (*P*<.10 and *P*>.05) and significance (*P*<.05). Separate *t* tests were conducted for variables in the full sample and for each group examined (cisgender heterosexual men, cisgender heterosexual women, and sexual minority men and women).

## Results

### Demographics

Of the 24 participants, 8 (33%) identified as cisgender heterosexual men, 9 (38%) as cisgender heterosexual women, and 7 (29%) as LGBTQ (1 [4%] identified as gay, 4 [17%] as bisexual, 1 [4%] as queer, and 1 [4%] as questioning). Of the 7 participants who identified as LGBTQ, 6 (86%) identified as female and 1 (14%) identified as male. The mean age of participants was 19.63 (SD 0.97) years, and most were in their second year of school. Most participants identified as White (16/24, 59%), non-Hispanic, or non-Latinx (15/24, 63%), and reported being in a long-term monogamous relationship lasting at least 6 months (10/24, 42%). Participants reported engaging in 2.08 (SD 2.04) episodes of heavy episodic drinking (≥4 drinks for individuals assigned female at birth; ≥5 drinks for individuals assigned male at birth) per month on average. Furthermore, participants reported drinking 4.75 (SD 4.87) drinks per week on average. Sample characteristics are presented in Table 1. Of the 30 initially enrolled, 2 (7%) cisgender heterosexual men, 1 (3%) cisgender heterosexual woman, and 3 (10%) LGBTQ participants did not fully complete the study procedures and were excluded from the analyses.

**Table 1:** Demographic characteristics of participants in open pilot.

Demographics			Cisgender heterosexual men (n=8)		Cisgender heterosexual women (n=9)		LGBTQ^a^ individuals (n=7)		Total sample (N=24)
**Racial identity, n (%)**									
	Asian	0 (0)		0 (0)		2 (29)		2 (8)	
	Black	1 (13)		0 (0)		1 (14)		2 (8)	
	White	7 (87)		7 (78)		2 (29)		16 (67)	
	Multiracial	0 (0)		2 (22)		1 (14)		3 (13)	
	Other	0 (0)		0 (0)		1 (14)		1 (4)	
**Ethnicity, n (%)**									
	Hispanic or Latinx	1 (13)		4 (44)		3 (43)		8 (33)	
	Non-Hispanic or non-Latinx	7 (88)		5 (56)		3 (43)		15 (63)	
	In sorority or fraternity	1 (13)		1 (11)		0 (0)		2 (8)	
**Relationship status, n (%)**									
	Not dating	3 (38)		2 (22)		0 (0)		5 (21)	
	Casually dating	2 (25)		2 (22)		5 (71)		9 (38)	
	Involved in a long-term monogamous relationship	3 (38)		5 (56)		2 (29)		10 (42)	
Age (years), mean (SD)			19.88 (1.13)		19.78 (0.97)		19.14 (0.69)		19.63 (0.97)
Years in college, mean (SD)			1.75 (0.87)		1.78 (0.97)		1.49 (0.79)		1.67 (0.87)
Heavy episodic drinking days per month, mean (SD)			3.00 (3.30)		1.56 (0.73)		1.71 (0.95)		2.08 (2.04)
Drinks per week, mean (SD)			7.12 (5.94)		3.77 (4.76)		3.28 (2.87)		4.75 (4.87)

^a^LGBTQ: lesbian, gay, bisexual, trans, queer or questioning.

### Usability and Acceptability of +Change

On average, participants took 17.52 (SD 11.75) minutes to complete *+Change*. Overall, the participants were satisfied with the information quality (mean 5.52, SD 1.19), interface quality (mean 5.98, SD 1.05), and system usefulness (mean 5.74, SD 1.05) of *+Change*. In terms of *+Change*’s helpfulness, participants reported they found the intervention content helpful (mean 5.22, SD 1.51) and believed their peers would as well (mean 5.17, SD 1.47).

### Alcohol Misuse

Results of *t* test analyses indicated that participants reported significant pre-post decreases in drinking norms, but did not report significant pre-post differences in injunctive drinking norms or in stages of change (Table 2).

**Table 2:** Changes in pre-post alcohol and sexual assault variables in open pilot.

Variables		Cisgender heterosexual men, mean (SD)			Cisgender heterosexual women, mean (SD)			LGBTQ^a^, mean (SD)			Total sample			
		Before *+Change*	After *+Change*	Before *+Change*		After *+Change*	Before *+Change*		After *+Change*	Before *+Change*, mean (SD)		After *+Change*, mean (SD)	*t* test (*df*)	*P* value
**Alcohol variables**														
	Descriptive norms	24.71 (16.50)	7.60 (3.78)	20.50 (9.21)		7.78 (6.16)	9.60 (3.36)		9.00 (0.00)	16.00 (7.01)		7.64 (5.24)	3.79 (20)	.002
	Injunctive norms	4.99 (0.80)	5.19 (1.03)	5.62 (0.79)		5.36 (1.14)	5.13 (0.75)		4.93 (0.51)	5.25 (0.79)		5.16 (0.90)	0.45 (20)	.66
	Contemplation ladder	.63 (1.06)	.75 (1.03)	4.33 (4.33)		5.13 (4.76)	.71 (1.89)		0.00 (0.00)	2.04 (3.33)		2.14 (3.65)	0.30 (22)	.77
**Sexual assault victimization variables**														
	Likelihood of incapacitated sex	1.13 (0.35)	1.00 (0.00)	3.13 (1.46)		2.43 (2.30)	4.14 (2.04)		3.14 (2.12)	2.76 (1.89)		2.19 (1.94)	1.49 (20)	.15
	Men’s victimization estimate	5.71 (3.15)	14.29 (6.07)	16.14 (18.43)		16.00 (7.59)	24.00 (16.36)		22.60 (15.93)	14.37 (15.23)		17.17 (10.11)	−.59 (17)	.56
	Women’s victimization estimate	21.71 (13.38)	49.00 (15.82)	39.43 (30.18)		44.67 (20.22)	45.00 (15.81)		46.20 (11.41)	34.37 (22.85)		46.78 (15.58)	−1.86 (17)	.08
	SGM’s^b^ victimization estimate	12.17 (13.79)	49.71 (12.41)	30.43 (25.88)		51.17 (25.99)	36.00 (14.75)		52.60 (12.40)	25.89 (21.18)		51.00 (17.05)	−3.95 (16)	.001
**Sexual assault perpetration variables**														
	Precontemplation	2.62 (0.59)	2.10 (0.71)	2.19 (0.66)		1.71 (0.52)	2.00 (0.58)		1.47 (0.51)	2.29 (0.64)		1.78 (0.61)	4.40 (19)	>.001
	Contemplation	2.19 (0.26)	2.71 (0.62)	2.97 (0.50)		3.00 (0.40)	2.94 (0.65)		3.14 (0.69)	2.59 (0.12)		2.94 (0.12)	−3.10 (19)	.006
	Rape myths	1.33 (0.13)	1.19 (0.11)	1.34 (0.33)		1.27 (0.25)	1.11 (0.04)		1.03 (0.02)	1.27 (0.23)		1.18 (0.19)	2.50 (21)	.02
	Estimated false reports	18.71 (16.83)	7.43 (7.91)	20.38 (22.17)		11.33 (8.36)	2.75 (1.50)		3.75 (1.50)	16.05 (18.34)		7.94 (7.39)	2.11 (15)	.05
	While drinking, decide not to have sex with someone who is drunk	4.89 (1.76)	5.38 1.19)	3.67 (1.97)		5.60 (0.55)	3.67 (1.97)		5.60 (0.55)	4.15 (1.69)		5.00 (1.48)	−2.20 (20)	.04
**Bystander variables**														
	Likelihood	4.07 (0.37)	4.29 (0.31)	4.09 (0.55)		4.23 (0.38)	4.24 (0.20)		4.55 (0.23)	4.12 (0.40)		4.34 (0.33)	−2.45 (21)	.02
	Efficacy	83.86 (9.64)	85.89 (15.71)	87.04 (9.15)		88.31 (7.88)	91.47 (6.44)		96.05 (4.33)	87.03 (8.82)		89.40 (11.02)	−1.51 (20)	.15

^a^LGBTQ: lesbian, gay, bisexual, trans, queer or questioning.

^b^SGM: sexual and gender minority.

### Sexual Assault Victimization Risk

Results of the *t* test analyses indicated that there was no significant pre-post difference in participants’ estimations of the risk of experiencing incapacitated sexual assault victimization themselves while in college (Table 2). However, all participants significantly increased their estimation of how many LGBTQIA+ students had experienced sexual assault since entering college significantly after *+Change* (Table 2).

### Sexual Assault Perpetration Risk

Results of the *t* test analyses indicated that after *+Change*, participants were significantly more aware of the problem of sexual assault on their campus (ie, less precontemplative) and had greater intentions to make changes to prevent sexual assault perpetration (ie, more contemplative; Table 2). Participants reported significant reductions in pre-post rape myths and increases in deciding not to have sex with someone who was drunk (Table 2). In relation to false reports, participants at baseline estimated that, on average, 15.76% (SD 17.83%) of sexual assaults at their university were false reports. After participating in *+Change*, participants estimated that, on average, 7.94% (SD 7.39%) of sexual assaults at their university were false reports. This decrease in estimated false reports was a trend that did not reach significance (*P*=.052).

### Bystander Intentions

Results of the *t* test analyses indicated that participants reported a significant preintervention to postintervention increase in the likelihood to intervene when witnessing sexual assault and nonsignificant increases in bystander efficacy following *+Change* (Table 2).

## Discussion

### Overview

This is the first program to provide personalized normative feedback to students who identify as LGBTQ and integrate multiple components of alcohol-related sexual assault prevention including victimization risk reduction, perpetration prevention, and bystander intervention training. Although a larger clinical trial to examine *+Change*’s efficacy is needed, these findings provide initial evidence that a comprehensive alcohol and sexual assault prevention program can be used among college students of varied genders and sexual orientations. Given the high rates of alcohol use and sexual assault among women and LGBTQ individuals, the rates of men as victims, and differences in perpetration rates, tailored content based on gender and sexual orientation are needed to move the prevention field forward.

### Principal Findings

The results supported the usability and acceptability hypotheses such that *+Change* had high usability and acceptability ratings among cisgender heterosexual women, cisgender heterosexual men, and LGBTQ college students. Furthermore, despite the relatively low power to test for significant differences, there were some significant initial indicators suggesting that *+Change* may be helpful.

The findings from this study suggest that *+Change* may be an acceptable strategy to target alcohol and sexual assault among cisgender heterosexual men, cisgender heterosexual women, and LGBTQ college students. Overall, participants rated *+Change* to be acceptable across several usability domains including information quality, interface quality, and system usefulness. Furthermore, participants indicated that *+Change* was helpful for themselves and believed it would be helpful for their peers. This may be because of the brief duration of the prevention program and the user-friendly format of the web-based personalized feedback intervention.

There was a significant decrease in descriptive drinking norms after participating in *+Change*. Although differences between cisgender heterosexual men, cisgender heterosexual women, and LGBTQ individuals were not examined because of the small sample size, mean values suggest that the largest changes may have occurred within cisgender heterosexual men and cisgender heterosexual women. Although there were no significant changes in injunctive drinking norms or in stages of change in drinking, an examination of the means before and after *+Change* suggests that there were small changes in the direction toward lower injunctive drinking norms and higher motivations for change. These findings are similar to other social norms interventions targeting drinking among college students [34] and suggest that targeting descriptive drinking norms is a viable strategy when targeting both alcohol and sexual assault among college students.

There were significant increases in the awareness of sexual assault victimization risk among LGBTQ students during college. Specifically, participants estimated that more LGBTQ students experienced sexual assault during college at their university after *+Change* than before *+Change*. This is an important finding because awareness of sexual assault perpetrated against LGBTQ students could encourage bystander intervention behavior if a potential sexual assault is witnessed against an LGBTQ peer. This is also important because 17 years is the median age at which LGBTQ individuals begin to identify as LGBTQ (Pew Research Center [59]). Therefore, although individuals are identified as cisgender and heterosexual at the time of the intervention, their identity may change later in life. There was also a similar nonsignificant trend among female students. Although group comparisons were not assessed, an examination of the means suggested that LGBTQ students estimated that all college students experienced sexual assault during college more than other groups. This may be because LGBTQ students themselves have higher rates of sexual assault [21] and therefore, may be more aware of the risk for all college students. Nonetheless, the findings suggest that providing current rates of risk based on gender identity and sexual orientation can change one’s perceived risk of experiencing sexual assault on a particular college campus.

Importantly, there were some indicators that *+Change* may have the potential to reduce sexual assault perpetration. Specifically, participants reported significantly less precontemplation and significantly more contemplation in terms of readiness to change sexual assault on their college campuses. In addition, participants reported decreased endorsement of rape myths and decreased estimate of how many sexual assault reports are false reports. Interestingly, before *+Change*, cisgender men and women believed that approximately one-fourth of reported sexual assaults at their university were false reports. This finding suggests that work is needed to change perceptions, given that only 5.9% of assaults are false accusations, the same rate as other crimes [60]. Furthermore, participants indicated increases in deciding not to have sex with someone who is drunk while they are drinking. This is an important behavioral intention indicator as it suggests that *+Change* may be helpful in reducing incapacitated sexual assault perpetration.

In terms of bystander intentions and attitudes, participants reported a significant increase in the likelihood to intervene when witnessing sexual assault and nonsignificant increases in bystander efficacy. These results are promising, especially in light of the theory suggesting that intentions to perform a behavior are the closest cognitive antecedent of behavioral performance [61,62]. Furthermore, both longitudinal [63] and experimental [64] studies have found that bystander intentions predict subsequent bystander behavior for sexual assault.

### Comparison With Prior Work

*+Change* is the first program to tailor content based on gender identity and sexual orientation. It is also the first program to integrate sexual assault victimization risk reduction, perpetration prevention, and bystander intervention training within one program. Therefore, this work extends previous research indicating that alcohol and sexual assault risks differ based on gender and sexual orientation [1-4], and previous calls for integrated programs for victimization risk reduction, perpetration prevention, and bystander intervention training [43]. Previous work has tested nontailored interventions of one component of sexual assault, such as the *Green Dot* which focuses on bystander intervention training, among sexual minority high-school students and found that the bystander content was not effective at reducing sexual assault among sexual minority youth [30]. Therefore, this study provides promising initial findings for an intervention that may address this large gap in prevention literature.

### Limitations

This study had several limitations including the fact that it was an open pilot study of a small sample of college students at one university. Therefore, conclusions on the initial outcomes are only preliminary and a large-scale randomized clinical trial across multiple universities is needed. In addition, as no participants identified as gender-diverse, future research is needed to assess the efficacy of *+Change* among gender-diverse students. As the assessments were conducted on the same day, before and after *+Change*, they were not able to capture any behavioral changes. Future efficacy trials should examine whether *+Change* is effective at reducing alcohol use, sexual assault victimization, perpetration, and increasing bystander intervention behaviors to determine efficacy. This study assessed potential helpfulness and distress in assessing acceptability. Future research should use more in-depth acceptability measures. Future research is needed to understand Black, Indigenous, and People of Color students and LGBTQ Black, Indigenous, and People of Color students, as their experiences may differ and they could benefit from tailored interventions. LGBTQ students were included within one group rather than including different intervention components for the LGBTQ subgroups. This is problematic as LGBTQ students are not a heterogeneous group. However, this is a necessary first step in tailoring the programs for LGBTQ students. Although it appears in this sample that the LGBTQ participants did not engage in more alcohol use than their cisgender heterosexual peers, which is likely because all participants enrolled in this study engaged in heavy episodic drinking at least once in the past month. Research findings that LGBTQ men and women engage in drinking at higher levels than their cisgender heterosexual peers do not include a restricted sample as in this study and this sample likely has a ceiling effect because of the inclusion criteria. Although this study is limited to the United States, research indicates that sexual assault in higher education occurs at high rates across the globe [65]. Furthermore, alcohol-involved sexual assault is a global problem [66]. Future research should focus on the development of integrated prevention programs for alcohol use and sexual assault using culturally appropriate content worldwide.

### Conclusions

*+Change* is the first program to integrate sexual assault victimization risk reduction, perpetration prevention, and bystander intervention training into one program. This is important to reduce both university costs and student time. It is also the first program to provide tailored content for LGBTQ students. This is important to acknowledge and address the unique risks of LGBTQ students as ignoring their needs may be a form of heterosexism embedded within the university prevention programming. Finally, it harnesses the strength of previous works [9,41]. The findings from this study suggest that *+Change* has high acceptability and usability among college students. Furthermore, there were several pre-post differences in outcomes related to alcohol use and sexual assault suggesting the need for a large randomized clinical trial.

## Appendix

Multimedia Appendix 1 Example positive change content.

## Abbreviations

LGBTQ: lesbian, gay, bisexual, trans, queer or questioning
LGBTQIA+: lesbian, gay, bisexual, trans, queer or questioning, intersex, and asexual
