# Supplementary material for: Web-Based Alcohol and Sexual Assault Prevention Program With Tailored Content Based on Gender and Sexual Orientation: Preliminary Outcomes and Usability Study of Positive Change (+Change)
Source: JMIR Form Res. 2022 Jul 22;6(7):e23823. doi: 10.2196/23823 (PMC9356330; doi:10.2196/23823)
Supplement: Multimedia Appendix 1 [file formative_v6i7e23823_app1.pdf]

Heavy episodic drinking

CHANGE

Theoretical Framework	Example Content
Social Norms Theory	"You have XX drinks per week. You think a typical student at [university] who identifies as [a man/a woman/LGBTQIA+[a]] has XX drinks per week. The typical student at [university] who identifies as [a man/a woman/LGBTQIA+] actually has about XX drinks per week."
Alcohol Myopia Theory	Personalized BAC[b] charts with sexual assault risk examples at each BAC (eg, ".08-.10 sexual judgement impaired, unable to interpret signs of sexual disinterest"), and psychoeducation provided on misperception of sexual interest while intoxicated because of cognitive impairment.
Alcohol Expectancy Theory	Personalized feedback on alcohol expectancies and sex-related alcohol expectancies paired with psychoeducation: "You said that the following effects related to drinking and sex are likely to happen: [insert sex-related alcohol expectancies endorsed on baseline survey]. Even though a lot of people might think that alcohol makes sex better, alcohol can actually decrease sexual pleasure and performance."
Minority Stress	Psychoeducation about how daily discrimination because of gender identity and sexual orientation can be associated with alcohol use and alcohol-related consequences among LGBTQIA+ students.
Skills Deficit Model	Psychoeducation on protective behavioral strategies to reduce the harm of alcohol use. Participants choose which strategies they would like to use in the future to reduce the harm of alcohol use.

[a]LGBTQIA+: lesbian, gay, bisexual, trans, queer/questioning, intersex, and asexual.
[b]BAC: blood alcohol concentration.

Sexual assault victimization risk reduction

CHANGE

Theoretical Framework	Example Content
Social Norms Theory	"You believe that XX% of students at [university] who identify as [a man/a woman/LGBTQIA+] who drink have been sexually assaulted. XX% of students at [university] who identifies as [a man/a woman/LGBTQIA+] have been sexually assaulted."
Alcohol Myopia Theory	Personalized BAC charts with sexual assault victimization risk examples at each BAC (eg, ".08-.10 judgement impaired, unable to perceive sexual assault victimization risk").
Assess, Acknowledge, Act	Personalized interactive psychoeducation and practice regarding sexual assault risk perception and sexual assault resistance strategies using cartoon scenarios in drinking situations. Participants choose the gender (man/woman/nonbinary) of the potential victim, potential perpetrator, and potential bystander in the scenarios. Participants are provided with feedback on their choice of resistance strategy and provided with options of how to respond to potential sexual assault situations (method previously developed and tested).
Cognitive Mediational Model	Personalized injunctive feedback for common barriers to using active sexual assault resistance strategies (eg, "XX% of students at [university] who identify as [a man/a woman/LGBTQIA+] reported that they would respect someone who strongly told an acquaintance to stop if they didn't consent to sex even if they were friends with them").

Sexual assault perpetration prevention

Theoretical Framework	Example Content
Social Norms Theory	“You believe that XX% of students at [university] who identify as [a man/a woman/LGBTQIA+] verbally ask for consent before sex. XX% of students at [university] who identifies as [a man/a woman/LGBTQIA+] verbally ask for consent before sex.”
Alcohol Myopia Theory	Personalized BAC charts with sexual assault risk examples at each BAC (eg, “.08-.10 sexual judgement impaired, unable to interpret signs of sexual disinterest”), psychoeducation provided on misperception of sexual interest while intoxicated because of cognitive impairment.
Integrated Model of Sexual Assault and Acquaintance Rape	Personalized interactive psychoeducation and practice regarding sexual assault perpetration risk, sexual consent, perceptions of false accusations, and practice using scenarios. Participants choose the gender (man/woman/nonbinary) of the potential victim, potential perpetrator, and potential bystander in the scenarios. Participants are provided with feedback on options of behaviors to engage in instead of perpetration.

Bystander intervention norms and skills training

CHANGE

Theoretical Framework	Example Content
Social Norms Theory	"XX% of students at [university] who identify as [a man/a woman/LGBTQIA+] reported that they would respect someone who steps in."<br>"XX% of students at [university] who identify as [a man/a woman/LGBTQIA+] would ask if everything was okay if they witnessed someone pressuring another person to leave with them."
Alcohol Myopia Theory	Personalized BAC charts with bystander intervention examples at each BAC (eg, ".08-.10 sexual judgement impaired, unable to interpret signs of a potential sexual assault situation among peers").
Integrated Model of Sexual Assault and Acquaintance Rape	Personalized interactive psychoeducation and practice regarding bystander intervention and practice using scenarios. Participants choose the gender (man/woman/nonbinary) of the potential bystander in the scenarios. Participants are provided with feedback on their choice of bystander intervention and options of other bystander behaviors to engage in.
Theory of Planned Behavior	Personalized feedback regarding attitudes, perceived norms, and perceived behavioral efficacy to intervene as a bystander in a potential sexual assault situation.
